# Na/K-ATPase Signaling and Cardiac Pre/Postconditioning with Cardiotonic Steroids

**DOI:** 10.3390/ijms19082336

**Published:** 2018-08-09

**Authors:** Pauline V. Marck, Sandrine V. Pierre

**Affiliations:** Marshall Institute for Interdisciplinary Research, Marshall University, Huntington, West Virginia, WV 25701, USA; marck@marshall.edu

**Keywords:** ouabain, digoxin, ischemia, reperfusion, acute myocardial infarction, kinase

## Abstract

The first reports of cardiac Na/K-ATPase signaling, published 20 years ago, have opened several major fields of investigations into the cardioprotective action of low/subinotropic concentrations of cardiotonic steroids (CTS). This review focuses on the protective cardiac Na/K-ATPase-mediated signaling triggered by low concentrations of ouabain and other CTS, in the context of the enduring debate over the use of CTS in the ischemic heart. Indeed, as basic and clinical research continues to support effectiveness and feasibility of conditioning interventions against ischemia/reperfusion injury in acute myocardial infarction (AMI), the mechanistic information available to date suggests that unique features of CTS-based conditioning could be highly suitable, alone /or as a combinatory approach.

## 1. Introduction

Without a doubt, the prompt restoration of blood flow to reinstate the perfusion of the ischemic myocardium has substantially improved the outcomes for patients hospitalized with acute myocardial infarction (AMI) [1]. It is also clear that life-saving reperfusion therapy is double-edged, as it ineluctably brings about the structural and functional damage of reperfusion injury to the myocardium [2]. With an estimated potential to reduce the size of the infarct by up to 40%, the development of clinically effective strategies to reduce reperfusion injury in AMI is one of the most-anticipated advances in cardiovascular therapies for the current decade [3]. To date, there is no stronger protection against reperfusion injury than the one afforded by adjunct conditioning treatment at reperfusion. As basic and clinical research continues to support the effectiveness and feasibility of conditioning interventions, this review covers insights into the protective cardiac Na/K-ATPase-mediated signaling triggered by ouabain and other cardiotonic steroids (CTS), as well as its potential application in the context of the enduring debate over the use of CTS in the ischemic heart.

## 2. Cardiac Pre- and Post-Conditioning against Ischemia/Reperfusion Injury

In 1986, Murry et al. first described the ability to precondition (PC) the heart to protect against infarction following ischemia/reperfusion injury (I/R). In dogs, the seminal study showed that a sequence of few very brief ischemic episodes induced by coronary occlusion, interspersed among brief periods of reperfusion, limited the cardiac damage induced by a subsequent prolonged ischemic insult. Strikingly, the infarct size was substantially smaller in the dogs that were exposed to the short periods of ischemia, than in the controls. The authors coined the term ischemic preconditioning (IPC) to describe this phenomenon [4]. Given its invasive nature and the need for intervention prior to the ischemic event, IPC’s limited clinical application was recognized early on. However, pre-clinical and clinical research has since shown that IPC-like protection can be obtained through ischemic post-conditioning (IPost) (applied at the time of reperfusion) and/or remote ischemic conditioning (RIC) (applied non-invasively to a limb during or after myocardial ischemia) [5].

## 3. Pharmacologically-Induced Cardiac Protection

**Figure 1:**
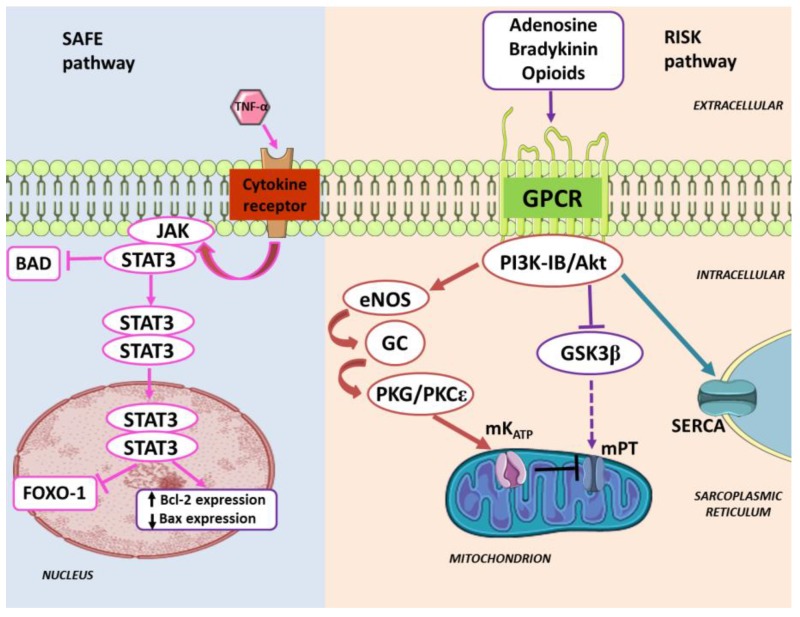
Major cardiac conditioning signaling pathways. The activation of cardioprotective signaling RISK, eNOS/PKG, and SAFE pathways by conditioning triggers is represented (modified from the literature [10]). BAD—Bcl-2-associated death promoter; Bax—Bcl-2-associated X protein; Bcl2—B-cell lymphoma 2; eNOS—endothelial nitric oxide synthase; FOXO-1—forkhead box protein O1; G—guanylyl cyclase; GPCR—G protein-coupled receptors; GSK3β—glycogen synthase kinase 3 beta; JAK—Janus kinase; mKATP—mitochondrial potassium ATP channel; mPT—mitochondrial permeability transition pore; PI3K-IB—phosphoinositide 3-kinase class IB; PKG—protein kinade G; PKCε—protein kinase C epsilon; RISK—reperfusion injury salvage kinase; SAFE—survivor activating factor enhancement; SERCA—sarco/endoplasmic reticulum Ca^2+^-ATPase; STAT3—signal transducer and activator of transcription 3; TNF-α—tumor necrosis factor alpha.

With the characterization of the cellular mechanisms involved in IPC, the concept of pharmacological PC took form [6]. Indeed, although PC was initially described as a response of the myocardium to ischemia, it soon became apparent that a similar phenotype could be elicited by other stimuli. Pharmacological strategies to activate IPC-like protective signal transduction pathways, while avoiding the vascular and myocardial injury that could result from coronary artery occlusion, were widely seen as less harmful, and thus more clinically suitable than the IPC-based strategies. A number of pharmacological agents, including agonists of G protein-coupled receptor (GPCR)s (adenosine A1 or A3, bradykinin B2, α1-adrenergic, muscarinic M2, angiotensin AT1, and endothelin, δ1-opioid), nitric oxide (NO) donors, phosphodiesterase inhibitors, and various noxious stimuli (such as endotoxin derivatives, various cytokines, and reactive oxygen species), have been found to elicit an IPC-like protection (reviewed in the literature [7]). Figure 1 summarizes the main signaling cascades that have been involved to date, including the eNOS/PKG, reperfusion injury salvage kinase (RISK) [8], and the survivor factor enhancement (SAFE) pathways, which ultimately result in the opening of the mitoK-ATP channel (mK_ATP_) and inhibition of the mitochondrial permeability transition pore (mPT) [9].

## 4. Enduring Challenges and Current Goals for Clinical Application of Conditioning

Although a variety of ischemia- and drug-based conditioning strategies have been tested, the results of the clinical studies for improving the patient outcomes have been largely disappointing. Beyond the need to refine the design of experimental and clinical studies [11], a now well-recognized core issue faced by basic scientists and clinicians alike is the influence of confounding risk factors. Indeed, frequent comorbidities such as diabetes or hypercholesterolemia, as well as co-medications [12] differentially alter the key elements of the cardioprotective signaling pathways (Figure 1), and consequently, the efficacy of a given tested PC intervention. As an approach to overcome this limitation, combination therapies targeting multiple non-redundant pathways are increasingly being explored [5]. In this context, the unique properties of the CTS signaling described in the following section represent a potential safe approach to consider in the protection against cardiac I/R injury.

## 5. Pre/Postconditioning with Cardiotonic Steroid through Cardiac Na/K-ATPase Signaling

CTS (digitalis in particular) have been used to treat heart failure for hundreds of years [13,14], long before the late Nobel Laureate Jens C. Skou uncovered their molecular target as the Na/K-ATPase [15,16,17]. Na/K-ATPase is the membrane-spanning enzyme complex that uses the energy of ATP hydrolysis for the coupled active transport of Na^+^ and K^+^ across the plasmalemma of mammalian cells [18,19,20]. CTS induce moderate inotropy by inhibiting sarcolemmal Na/K-ATPase, which raises the intracellular Na^+^ and Ca^2+^ through the Na^+^/Ca^2+^-exchanger, and subsequently increases myocardial contractility [21,22]. Alteration of cardiac Na/K-ATPase has long been recognized as a key aspect of I/R pathophysiology [23,24], which contributes to cardiomyocyte demise through mechanisms that go far beyond a simple disruption of Na^+^ and Ca^2+^ homeostasis secondary to ATP depletion, and remains incompletely understood [25,26,27,28,29,30,31]. Clinically, interest in CTS for the management of acute myocardial infarction sparked early in modern cardiology, before molecular knowledge of Na/K-ATPase function in health and I/R became available [32]. Somewhat surprisingly, in the context of the molecular mechanism described above, experimental and clinical reports still suggested beneficial effects of an “appropriate and judicious” use of the CTS digitalis in patients with failing myocardium associated with acute myocardial infarction. There were also concerns over increased sensitivity to inotropic and toxic effects, and under the principle of “*primum non nocere*”, the prevalent message in the clinical arena has persisted as “there is no role for the prophylactic use of digitalis in the uncomplicated myocardial infarction” [33].

**Figure 2:**
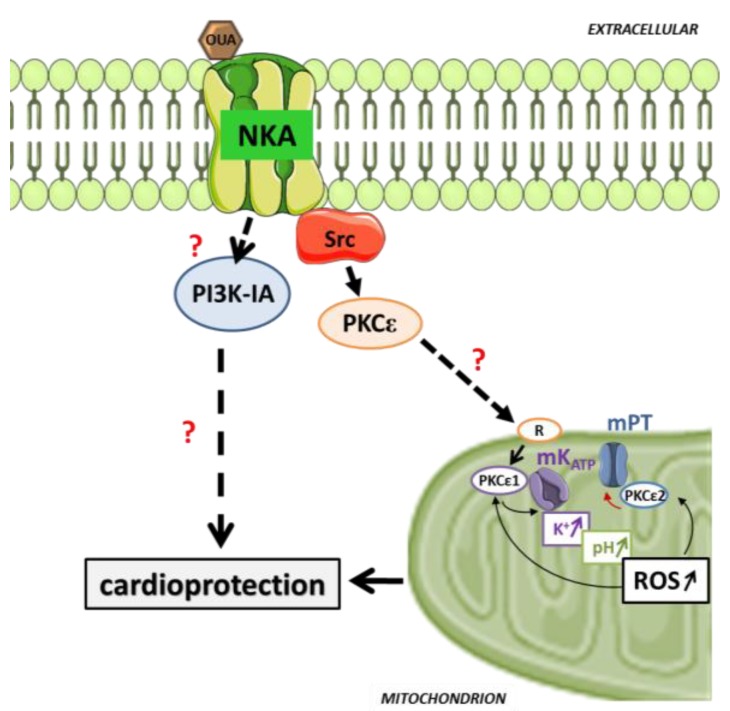
The ouabain preconditioning signaling pathway. Na/K-ATPase/Src activation precedes PKCε activation and translocation. An intramitochondrial pathway involves PKCε activation (PKCε1) and mKATP channel opening as a functional complex to trigger an increase in K^+^-uptake in the mitochondrial matrix. The mKATP-dependent matrix alkalinization is crucial in intramitochondrial signaling, leading to ROS production, which activates the second PKCε, PKCε2. PKCε2 inhibits the mitochondrial permeability transition pore(mPT) in a phosphorylation-dependent reaction. Furthermore, PKCε1 sustains the open state of mKATP channel through mKATP-dependent ROS activation. In addition to this mitochondrial cardioprotective signaling, ouabain-induced PI3K-IA activation is required for protection by OPC. The inhibition of either PKCε or PI3K-IA blunts the OPC-induced protection. mKATP—mitochondrial potassium ATP channel; mPT—mitochondrial permeability transition pore; NKA—Na/K-ATPase; OUA—ouabain; PKCε—protein kinase C epsilon type; PLC-γ—phospholipase C gamma; PI3K-IA—phosphoinositide 3-kinase class IA; ROS—reactive oxygen species; Src—proto-oncogene tyrosine-protein kinase.

Unsurprisingly, given such inauspicious pharmacological and clinical circumstances, the CTS pre/postconditioning hypothesis was not formulated until the 20 year-old discovery of Na/K-ATPase signaling, recognized in this special issue, came about. Indeed, it is the discovery of elements of the molecular signature of the CTS-induced signaling through the cardiac Na/K-ATPase that revealed striking similarities with those of the ischemic and pharmacological PC, and prompted further investigation. Specifically, by early 2000, it had become clear that exposure to the CTS ouabain triggers Src, protein kinase C epsilon (PKCε), ERK, mK_ATP_, and mitochondrial reactive oxygen species (ROS) production in the cardiac tissue [34,35,36]. Collectively, these represented a hallmark of the RISK pathway, which is common to most pharmacological preconditioners known at the time (Figure 1). Two studies specifically tested the hypothesis that exposure to the CTS ouabain could trigger preconditioning, and uncovered the first key characteristics in Langendorff-perfused rat heart preparations. In Pierre et al. [37], transient exposure to a subinotropic concentration of ouabain and wash-out prior to ischemia/reperfusion induced a structural and functional protection comparable to that observed with IPC in this model. By analogy to IPC, this phenomenon was termed Ouabain PreConditioning (OPC). Pharmacological inhibition further revealed that OPC requires both Src and PKCε activities, and that Src is required for PKCε activation. In Pasdois et al. [38], an alternate protocol consisting of a continuous exposure to increasing concentrations of ouabain also triggered OPC. Mechanistically, the study demonstrated the requirement for mK_ATP_-opening and ROS production, and it also revealed that OPC is independent of protein kinase G (PKG) and guanylyl cyclase (GC) activation, contrary to bradykinin-induced PC. This independence from PKG/GC was of particular interest, not only because it was the first indication that a unique mechanism of protection could set CTS apart from other pharmacological triggers of PC, but also because it indicated that CTS-induced PC signaling and inotropy relied on distinct mechanisms. Hence, although mK_ATP_-opening and ROS production are required for both OPC and positive inotropy, GC and PKG are required only for the latter. Collectively, these two studies also revealed that ouabain inotropy and OPC have distinct dose-dependence curves, and illustrated that although inotropy stops when ouabain is removed, OPC protection occurs even after ouabain is withdrawn, consistent with the general model of persistence of cardioprotective signaling cascades observed after washout of their triggers. Subsequently, D’Urso et al. successfully triggered CTS PC using the FDA-approved digoxin [39], indicating potentials in the clinical setting. Additionally, Morgan et al. [40] reported that CTS-induced PC protection could be achieved in the rabbit heart, a model that recapitulates human myocardial physiology, vulnerability to ischemic injury, and CTS pharmacology better than the rat or mouse heart [35,40,41,42,43,44]. The lower ouabain concentration (nM range) [40] correlated with the higher ouabain affinity of the Na/K-ATPase α1 isoform in the rabbit species compared to the rat, suggesting a key role of α1 in CTS PC signaling. As summarized in Figure 2, OPC signaling includes an intramitochondrial signaling pathway, common to most if not all forms of PC [45,46,47]. It involves at least two mitochondrial PKCε in the sequence that leads to a mK_ATP_-opening, production of ROS, and inhibition of mPT. Remarkably, activation of the Na/K-ATPase cardioprotective signaling pathway by OPC protects the myocardial Na/K-ATPase enzyme function itself against I/R [29,48], a feature that was also noted in IPC [49]. Several aspects of OPC signaling are unique, and contrast with IPC and GPCR-based forms of pharmacological preconditioning (Figure 1). Firstly, as mentioned earlier, it is a cGMP-independent pathway, in contrast to numerous major forms of cardioprotection [50]. Secondly, it is mediated by PI3K-IA (rather than PI3K-IB), in parallel rather than upstream from the PKCε activation [51]. The reliance on PI3K-IA rather than IB in the pre-ischemic phase (as observed for IPC or adenosine) is a rare occurrence in known pharmacological PC, and suggests that OPC could trigger “insulin-like” protective effects related to the substrate utilization or cell survival. Studies have also shown that the PI3K-IA activation is highly protective at reperfusion [52]. Finally, and perhaps even more surprisingly (yet consistent with independence from the cGMP pathway) is the lack of requirement for Akt activation in OPC [51]. Potentially, these unique mechanistic features could make a CTS-based approach very suitable, alone or in combination, for PC in patients whose disease and/or treatment may have altered cGMP, PI3K-IB, and/or Akt pathways. Therefore, we recently tested a more clinically applicable CTS-based protocol by comparing very low doses of ouabain and digoxin’s protective effects when given as a bolus at reperfusion, following 40 min of zero flow ischemia in Langendorff-perfused mouse heart preparation. The results showed that Na/K-ATPase cardioprotective signaling activation, increased cell survival, and improved functional recovery as effective as those obtained with IPostC can be obtained using digoxin [53].

## 6. Prospect and Future Directions

The first reports of cardiac Na/K-ATPase signaling [54,55,56], 20 years ago, have opened several major fields of investigation into the cardioprotective action of CTS drugs given at low doses, particularly in hypertrophy [57,58], as well as PC, as reviewed here. The mechanistic information available on PC suggests distinct features of CTS-based PC that could make this modality clinically relevant, alone or as a combinatory approach. There are also a number of remaining gaps and questions in our current knowledge of the pathway and its connection to other modalities of PC. For instance, the role of the key players of cell metabolism/survival Bcl2/Bax, ERK1/2, and GSK3-β, which are fixtures of PC signaling (Figure 1) and have been shown to be modulated by CTS/Na/K-ATPase signaling [54,59,60,61], remain to be tested on CTS-based PC.

As studies continue to explore the mechanism and efficacy of CTS conditioning, *in vivo* investigations in pre-clinical rodent and non-rodent models of AMI will be critical. Indeed, important aspects of the complex pathophysiology of I/R injury, such as sterile inflammation and cardiac/vascular remodeling, cannot be adequately evaluated *ex vivo*. Fundamentally, and given the proposed role of the Na/K-ATPase non ion-pumping function in cardiac myocyte survival after I/R injury [29], those models could also redefine the role of Na/K-ATPase signaling and endogenous CTS in the pathophysiology of I/R injury. Indeed, a role for endogenous CTS as hormones with distinct but related modulatory effects on cardiovascular homeostasis has been suggested in AMI and other physiological and pathophysiological conditions, such as pregnancy, exercise, salt-loading, or heart failure) [62,63,64,65,66], and could include a PC-based effect. In the context of I/R injury, the CTS release from the rat myocardium has been observed *ex vivo* within a short (15 min) ischemia [67], suggesting that the ischemia-induced release of CTS may occur during IPC. Ouabain, and potentially other CTS, could therefore be added to the list of paracrine/autocrine factors that are released during preconditioning ischemia and trigger protection by binding to their respective receptors. Some of the most promising cardioprotective candidate drugs to date, opioid and cannabinoid receptor agonists [68,69] or adenosine receptor agonists [70], belong to this category.
